# Large-Scale Brain Networks Supporting Divided Attention across Spatial Locations and Sensory Modalities

**DOI:** 10.3389/fnint.2018.00008

**Published:** 2018-02-27

**Authors:** Valerio Santangelo

**Affiliations:** ^1^Department of Philosophy, Social Sciences & Education, University of Perugia, Perugia, Italy; ^2^Neuroimaging Laboratory, Santa Lucia Foundation, Rome, Italy

**Keywords:** divided attention, frontoparietal, central executive, salience, network, independent component analysis (ICA), Granger, causality

## Abstract

Higher-order cognitive processes were shown to rely on the interplay between large-scale neural networks. However, brain networks involved with the capability to split attentional resource over multiple spatial locations and multiple stimuli or sensory modalities have been largely unexplored to date. Here I re-analyzed data from Santangelo et al. (2010) to explore the causal interactions between large-scale brain networks during divided attention. During fMRI scanning, participants monitored streams of visual and/or auditory stimuli in one or two spatial locations for detection of occasional targets. This design allowed comparing a condition in which participants monitored one stimulus/modality (either visual or auditory) in two spatial locations vs. a condition in which participants monitored two stimuli/modalities (both visual and auditory) in one spatial location. The analysis of the independent components (ICs) revealed that dividing attentional resources across two spatial locations necessitated a brain network involving the left ventro- and dorso-lateral prefrontal cortex plus the posterior parietal cortex, including the intraparietal sulcus (IPS) and the angular gyrus, bilaterally. The analysis of Granger causality highlighted that the activity of lateral prefrontal regions were predictive of the activity of all of the posteriors parietal nodes. By contrast, dividing attention across two sensory modalities necessitated a brain network including nodes belonging to the dorsal frontoparietal network, i.e., the bilateral frontal eye-fields (FEF) and IPS, plus nodes belonging to the salience network, i.e., the anterior cingulated cortex and the left and right anterior insular cortex (aIC). The analysis of Granger causality highlights a tight interdependence between the dorsal frontoparietal and salience nodes in trials requiring divided attention between different sensory modalities. The current findings therefore highlighted a dissociation among brain networks implicated during divided attention across spatial locations and sensory modalities, pointing out the importance of investigating effective connectivity of large-scale brain networks supporting complex behavior.

## Introduction

It is by now well established that higher-order cognitive processes rely on the interplay between large-scale neural networks (Bressler and Menon, 2010; Raichle, 2015; Wig, 2017). However, how such multiple networks interact to support a complex cognitive process such as divided attention is largely unknown. Divided attention consists in the capability to monitor and select multiple information at the same time (Jans et al., 2010). Previous research has demonstrated that monitoring of multiple streams of information typically results in a decrement of processing efficacy (Shaw and Shaw, 1977; Eriksen and St. James, 1986; Castiello and Umiltà, 1990, 1992; Müller M. M. et al., 2003; Müller N. G. et al., 2003). At a neurophysiological level, divided attention was shown to recruit high-level brain regions, such as the dorsal frontoparietal attention network (Fagioli and Macaluso, 2009, 2016; Santangelo et al., 2010), showing modulatory effects on sensory cortices deputed to process the incoming, multiple information (McMains and Somers, 2004, 2005; Sreenivasan et al., 2014).

However, one limitation of the previous studies devoted to the understanding of the neural correlates of divided attention relies on the use of subtraction paradigms that, by definition, are not sensitive to the co-activation of distinct networks (Friston et al., 1996). As such, less is known to date about whether the neurophysiological underpinnings of divided attention involve a dynamical interplay between multiple networks. Based on the re-analysis of previous data (Santangelo et al., 2010, reporting standard fMRI analyses), the current study aims at highlighting large-scale brain networks involved with divided attention, and more specifically, with divided attention across different spatial locations vs. different stimuli and sensory modalities. Attentional resources can be employed to monitor for a given stimulus that might appear from different locations (divided attention across multiple locations) or to monitor for different stimuli originating from the same spatial locations (divided attention across multiple stimuli). In this latter case, the stimuli to be monitored might come from the same or different sensory modalities, e.g., visual or auditory.

Previous research (Fagioli and Macaluso, 2009) reported that the dorsal frontoparietal attention network (encompassing the frontal eye-fields (FEF) and the intraparietal sulci (IPS), bilaterally) activated both when subjects divided attention across different spatial locations and across simultaneously presented visual stimuli (e.g., geometrical shapes of different colors). Fagioli and Macaluso (2009) interpreted these findings as suggesting a key role played by the dorsal frontoparietal network both for spatial and non-spatial divided attention. This might also indicate the existence of an interplay between divided attention and working memory, with increased working memory load when trying to monitor multiple visual stimuli at different locations, supported by frontoparietal regions (e.g., see for review Smith and Jonides, 1999; D’Esposito, 2001; see also Johnson and Zatorre, 2005, 2006; Johnson et al., 2007). However, following research (Santangelo et al., 2010) showed that dividing attention between two sensory modalities depended on the spatial distribution of attention, unlike monitoring two stimulus categories in the visual unisensory context. In fact, the behavioral cost of monitoring two vs. one sensory modality was shown to decrease when paying attention to two vs. one hemifield. These findings suggest that there is higher interference (greater behavioral costs) in monitoring two independent sensory modalities when attention is focused on a given spatial location as compared to multiple spatial locations. Overall, this suggests greater availability of processing resources when attending for different sensory modalities at different spatial locations. The more efficient performance in monitoring two modalities at separated locations compared to the same location was found to be supported by the posterior nodes of the dorsal frontoparietal network, namely the posterior parietal cortex, which might provide additional processing resources under this condition (Santangelo et al., 2010). This specific interplay between spatial- and sensory-related factors might indicate that—at least in multisensory contexts—divided attention across different spatial locations or sensory modalities might be subserved by different brain networks.

In the current study participants were asked to monitor streams of visual and/or auditory stimuli in one or two spatial locations for detection of occasional targets. This experimental design allowed contrasting conditions in which participants monitored one stimulus/modality (either visual or auditory) in two locations, i.e., the left and right hemifields (“att2loc”) vs. conditions in which participants monitored two stimuli/modalities (both visual and auditory) in one location (“att2mod”). The analysis of the independent components (ICA) was used to highlight any large-scale brain network operating during divided attention across spatial locations vs. sensory modalities. Based on the existent literature (Fagioli and Macaluso, 2009, 2016; Santangelo et al., 2010; Santangelo and Macaluso, 2013a), there might be expected a main involvement of the frontoparietal network during divided attention tasks, but also brain differences related to the specific interplay between spatial- and sensory-related factors in multisensory contexts (see, Santangelo et al., 2010) during divided attention over different spatial locations or sensory modalities. To investigate the specific contribution of regions within the ICs supporting either divided attention across spatial locations or sensory modalities Granger Causality Analysis (GCA) was employed. This allowed to assess any causal relationships (i.e., the effective connectivity) among the main nodes of the emerging ICs.

## Materials and Methods

### Participants

Thirteen right-handed healthy volunteers took part in the study. All participants had normal hearing and normal or corrected-to-normal visual acuity (with contact lenses). Because of poor accuracy on the task (<75%), one participant was excluded from statistical analysis, leaving 12 participants (6 males, age range: 20–33 years, mean age: 25.2 years). The independent Ethics Committee of the Santa Lucia Foundation (Scientific Institute for Research Hospitalization and Health Care) approved the study. Participants gave written informed consent before their participation.

### Stimuli and Task

**Figure 1:**
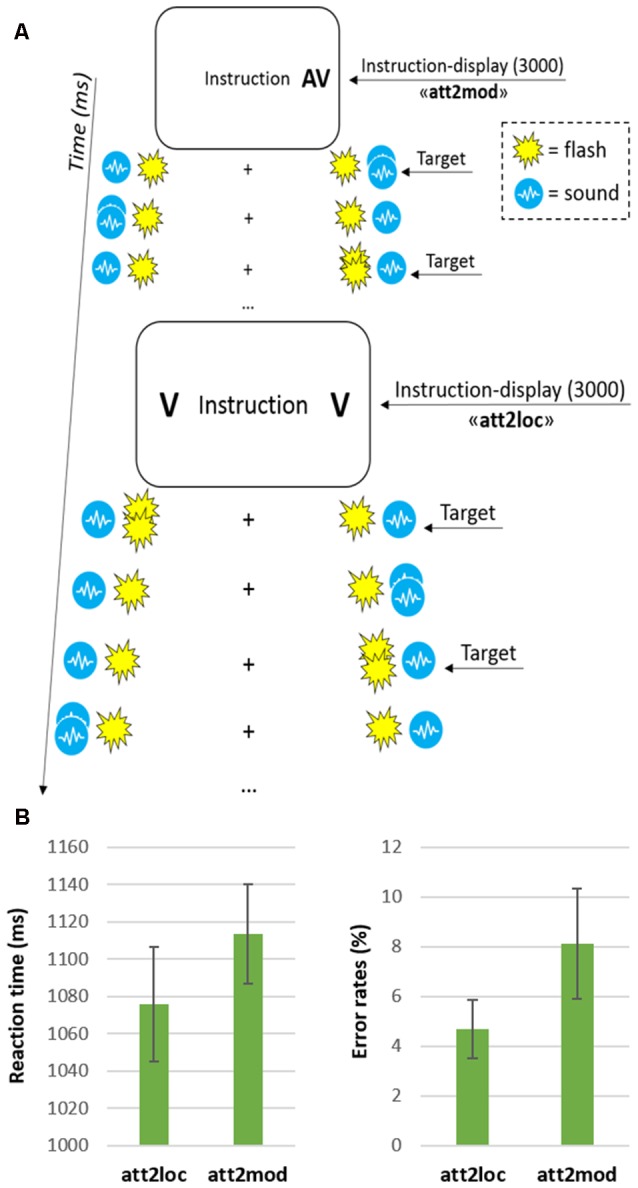
**(A)** Schematic diagram showing from top to bottom an example of a few trials in the “attending two modalities” (att2mod) and “attending two locations” (att2loc) conditions. Each block consisted of 10 trials and began with an instruction display signaling the current task. On each trial, the stimulation was always bilateral with two independent audiovisual streams on each side. Depending on the current condition, participants monitored one or two of the four sensory streams in one or two hemifields, responding to double pulses (i.e., the targets) in the relevant stream/streams while ignoring all other stimuli; **(B)** Reaction times (left graph) and error rates (right graph) for the two main conditions (att2loc and att2mod). The error bars represent the standard error of the means.

Stimuli and task were fully described in Santangelo et al. (2010), where we performed standard univariate analyses of this data set. Briefly, the participants’ task was to detect visual and/or auditory targets (i.e., one or two target modalities) in either or both hemifields (i.e., one or two target locations). During fMRI scanning, participants viewed a central display via a mirror system (see Figure 1A). Through the display, participants were presented with visual instructions regarding the upcoming attention task and with a central fixation cross (1.2° × 1.2°), which was displayed throughout the task. During the task participants were present with four simultaneous streams of stimuli, that is, a visual and an auditory stream on each hemifield. Two rubber pipes conducting sounds (i.e., bursts of white noise, sound pressure level, SPL = 115 dB) from two loudspeakers placed outside the MR room were used to present the auditory stimuli. The pipes were horizontally aligned with the fixation cross and connected to the left and right side of the coil. Participants were presented with either single (duration = 160 ms) or double bursts (160 ms on, 160 ms off, 160 ms on), with the latter serving as to-be-detected targets, when presented in the to-be-attended auditory stream. Optical fibers (diameter = 1 mm) connected with yellow light emitting diode (LED; luminance = 30 cd/m^2^) were instead used to present visual stimuli. Each optical fiber was located into a rubber pipe: this procedure allowed delivering visual and auditory stimuli from approximately the same locations, that is, around 30° to the left and right of central fixation point. As for the auditory stimuli, single (duration = 160 ms) and double flashes (160 ms on, 160 ms off, 160 ms on) were presented, with the latter serving as targets when delivered in the to-be-attended visual stream. Notably, the amount of stimulation was identical on both hemifields, with visual and auditory stimuli presented concurrently, regardless of the selective attention task that participants had to carry out.

The four simultaneous streams were presented in blocks of 25 s, corresponding to 10 consecutive trials. At the beginning of each block an instruction display indicating the location(s) and modality(ies) to be monitored was presented to the participants. The instruction display (duration = 3 s) included the text string “Instruction”, plus one or two letters on the left and/or right side (“V” for “monitor vision” and “A” for “monitor audition”). The arrangement of letter(s) and position(s) provided the relevant side and modality to be monitored in that block of trials (see Figure 1A). According to the instruction, the participants performed one of four possible attention tasks: (1) attend to one single modality in one hemifield, that is, detect either visual or auditory target in either the left or the right hemifield; (2) attend to one single modality in both hemifields, that is, detect either visual or auditory targets in both the right and left hemifield; (3) attend to both modalities in the same hemifield, that is, detect both visual and auditory targets in either the left or right hemifield and (4) attend to one modality in one hemifield, and the other modality in the opposite hemifield, that is, detect visual targets in the left hemifield and auditory targets in the right hemifield or vice versa. According to the main aim of the current study, here we directly compared task 2 vs. task 3, involving, respectively, “divided attention across two spatial locations” (att2loc) and “divided attention across two sensory modalities” (att2mod).

Participants task consisted in the detection of visual (double flashes) and/or auditory (double bursts) targets in one of the currently attended streams, while ignoring all the stimuli in the currently irrelevant streams. Participants signaled target detection by pressing a response button with their right index-finger. Each block included 10 trials, which started 2000 ms after the offset of the instruction display. Every 2500 ms a new trial started. The sequence of trials entailed two constraints: First, two target stimuli were never presented in the same trial when participants had to monitor for multiple streams. Second, there were always five targets in each currently relevant stream. Participants underwent four fMRI runs, each lasting about 6 min. Each run included 12 blocks, i.e., four tasks repeated three times. Overall, each participant was therefore presented with an amount of 480 trials, that is, 120 repetitions for each of the four attention tasks.

### fMRI Methods

#### Image Acquisition

A Siemens Allegra (Siemens Medical Systems, Erlangen, Germany) operating at 3T and equipped for echo-planar imaging (EPI) was used to acquire the functional magnetic resonance images. A quadrature volume head coil was used for radio frequency transmission and reception. Head movement was minimized by mild restraint and cushioning. Thirty-two slices of functional MR images were acquired using blood oxygenation level-dependent imaging (3 × 3 mm, 2.5 mm thick, 50% distance factor, repetition time = 2.08 s, time echo = 30 ms), covering the entirety of the cortex.

#### Image Processing

I used SPM12 (Wellcome Department of Cognitive Neurology) implemented in MATLAB R2012b (The MathWorks Inc., Natick, MA, USA) for data preprocessing and GLM. Each participant underwent four fMRI-runs, each comprising 477 volumes. After having discarded the first four volumes of each run, all images were corrected for head movements. Slice-acquisition delays were corrected using the middle slice as reference. All images were normalized to the standard SPM12 EPI template, resampled to 2 mm isotropic voxel size, and spatially smoothed using an isotropic Gaussian kernel of 8 mm FWHM. Time series at each voxel for each participant were high-pass filtered at 220 s and pre-whitened by means of autoregressive model AR(1).

#### Independent Component Analysis

The main aim of the current study was to highlight any large-scale brain network involved with divided attention across multiple spatial locations and sensory modalities. These brain networks were identified by the ICA. ICA is a blind-source computational method for separating a multivariate signal into additive subcomponents. The main assumption is that each subcomponent is statistically independent from each other. ICA was here implemented by means of the “Group ICA of fMRI Toolbox” (GIFT; Calhoun et al., 2001, 2009). This method involves performing ICA on functional data concatenated over every participant, creating a series of spatial maps and associated time courses for the group. The number of components was estimated to be 24 using the minimum description length criteria. The infomax algorithm was repeated 20 times with randomly initialized decomposition matrices and the same convergence threshold using ICASSO approach in GIFT (Himberg et al., 2004). As a finite set of data never result in exactly the same ICA model, ICASSO was introduced to estimate the overall reliability of the generated components. Back reconstruction was then used to create individual time courses and spatial maps from each participant’s functional data. Based on the spatio-temporal characteristics of each identified independent component (IC), 12 components reflecting noise were discarded after careful visual inspection (see Supplementary Figure S1 and Beckmann, 2012), leaving 12 components for further analyses.

#### Identification of Independent Components Related to Divided Attention

ICs with time courses related to the experimental design were identified using multiple regressions and the temporal sorting feature of the GIFT toolbox. Individual performance at the two main attention tasks was modeled with SPM12. Single subject models comprised two regressors, one including the onsets of “att2loc” blocks and the other including the onsets of “att2mod” blocks, plus a covariate of no interest including the onsets of the remaining blocks of trials (i.e., task 1: Attend to one single modality in one hemifield, and task 4: Attend to one modality in one hemifield, and the other modality in the opposite hemifield). All blocks were modeled with a duration of 25 s, i.e., 10 trials by 2500 ms each, time locked at the onset of the first trial of the block. The onsets of instruction displays were also included in the multiple regression model as covariates of no interest, with a duration of 5 s. All predictors were convolved with the SPM12 hemodynamic response function.

We tested the significance of the component time courses by doing statistics on the beta weights obtained after the temporal sorting, using the “Stats on Beta Weights” GIFT utility. Specifically, this utility allowed to assess the temporal fitting of the time course of a given IC with the events modeled with SPM. In accordance with the main aim of the current study, we assessed which component was involved with divided attention across spatial locations, by testing “att2loc > att2mod”, and with divided attention across sensory modalities, by testing “att2mod > att2loc”, by means of two-tailed paired *t*-tests. Holm-Bonferroni’s correction was applied to account for the risk of increased false positives as a function of an increased number of ICs tested (Gaetano, 2013). This procedure enabled to highlight two different brain networks, one operating during divided attention across space (IC 8), and the other operating during divided attention across sensory modalities (IC 15; see Table 1).

**Table 1:** Two-tailed paired *t*-tests assessing the involvement of independent components (ICs) with “att2loc > att2mod”, denoted by positive *t*-values, and with “att2mod > att2loc”, denoted by negative *t*-values, showing the involvement of IC 8 and IC 15, respectively.

IC	*t*-value	*p*-value
2	2.11	0.295
3	−1.64	0.427
5	−2.77	0.163
7	3.12	0.098
8	4.59	0.008
9	−1.76	0.427
11	2.45	0.225
13	−0.95	0.693
14	2.33	0.240
15	−5.79	0.001
18	0.98	0.693
23	−2.67	0.175

*P-values are corrected by Holm-Bonferroni’s procedure for multiple comparisons*.

#### Granger Causality Analysis

The effective connectivity among the main nodes of ICs supporting divided attention across spatial locations and sensory modalities was then assessed. While “functional” connectivity allows assessing the co-variation between two or more neurophysiological measures (i.e., time-series) without providing any information about directionality or causality, “effective” connectivity allows investigating which time-series is causing which other, thus drastically reducing the possibility of alternative interpretations (Stephan and Roebroeck, 2012). To investigate the effective connectivity among IC nodes GCA was used. CGA has the advantage with respect to other effective connectivity measures (e.g., dynamic causal modeling, DCM) of not requiring any* a priori* knowledge about within-network connectivity (Friston et al., 2013).

CGA is based on the notion that, if a time-series “X” causes a time-series “Y”, then knowledge of X should improve the prediction of Y more than information already in the past of Y. GCA allows computing causality by comparing the variance of the residuals after an autoregressive (AR) application to the reference signal Y, with the same variance obtained when autoregression is evaluated by combining both the past values of the signal Y and the past values of the potentially causing signal X. Previous literature has already demonstrated that CGA is a viable technique for analyzing fMRI time-series (Barnett and Seth, 2011; Seth et al., 2013; Wen et al., 2013) and that provides results that are consistent with other effective connectivity measures (i.e., DCM; Bajaj et al., 2016). We therefore modelled directional causality among multiple time series using GCA, as implemented in the “Multivariate Granger Causality Toolbox” (MVGC; Barnett and Seth, 2014; see also Seth, 2010).

**Figure 2:**
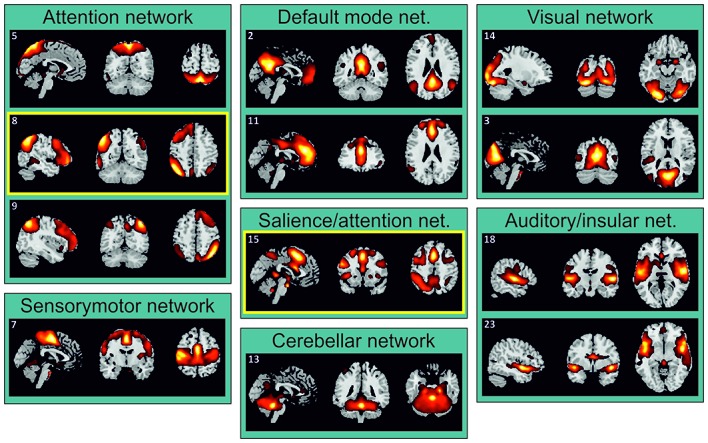
Task-related independent components (ICs) grouped according to their functional similarity. IC 8 and IC 15, supporting divided attention across space and sensory modalities, respectively (see Table 1), were highlighted by yellow boxes.

As showed in Figure 2, the IC 8 that was selectively involved during divided attention across spatial locations (att2loc > att2mod) included bilateral regions of the posterior parietal cortex and left regions of prefrontal cortex (see “Results” section below). On the other hand, IC 15 that was selectively involved with divided attention across sensory modalities (att2mod > att2loc), included both regions belonging to the dorsal frontoparietal network and regions belonging to the salience network. Accordingly, these areas (see Table 2) were selected as regions of interest (ROI) for the GCA. Each ROI consisted of a sphere (diameter = 8 mm, matching the FWHM of the smoothing filter) centered on the peak of activity of the regions belonging to IC 8 and IC 15 (Table 2). MarsBar SPM toolbox (v. 0.44) was used to extract and average time series from single subject designs within each ROI. Holm-Bonferroni correction was applied to account for the risk of increased false positives as a function of an increased number of comparisons across the nodes of the ICs tested.

**Table 2:** MNI coordinates of areas selected as regions of interest (ROI) in the IC 8 and IC 15, supporting divided attention across space and sensory modalities, respectively.

	Divided attention across space (IC 8)		
Area	*x*	*y*	*z*
L DLPFc	−50	10	30
L VLPFc	−56	18	8
L IPL	−48	50	54
R IPL	46	−48	52
L Ang	−42	−66	48
R Ang	54	−60	38
	**Divided attention across modalities (IC 15)**		
**Area**	***x***	***y***	***z***
ACC	−2	14	42
L aINS	−30	24	−4
R aINS	36	22	−2
L FEF	−28	2	58
R FEF	30	2	58
L IPS	−34	−48	46
R IPS	34	−46	48

*The time courses of the bold signal originated from these ROIs were used for the Granger causality analysis. Note: DLPFc, dorsolateral prefrontal cortex; VLPFc, ventrolateral prefrontal cortex; IPL, inferior parietal lobule; Ang, angular gyrus; ACC, anterior cingulate cortex; aIC, anterior insular cortex; FEF, frontal eye-fields; IPS, intraparietal sulcus*.

## Results

### Behavioral Data

The analysis of the behavioral performance—illustrated in Figure 1B—revealed that participants made more errors (including both false alarms and missed responses) when monitoring two sensory modalities (mean = 8.1%) compared with two spatial locations (4.7%; two-tailed paired *t*-test: *t*_(11)_ = 2.9, *p* = 0.015). Despite only marginally significant, the RT data were consistent with the latter finding, with slower target detection when participants monitored two sensory modalities (1114 ms) compared with two spatial locations (1076 ms; *t*_(11)_ = 2.1, *p* = 0.059; see, for extended behavioral results, Santangelo et al., 2010).

### fMRI Data

The ICA identified 24 ICs. Twelve of these ICs were unnoisy and related to the main attention tasks (Figure 2). Each of these ICs was attributed to a particular network on the basis of the previous literature (e.g., Damoiseaux et al., 2006; Shirer et al., 2012). These components were further analyzed in order to highlight which of them underlies divided attention across spatial locations and divided attention across sensory modalities.

#### Divided Attention across Spatial Locations: ICA and GCA

**Figure 3:**
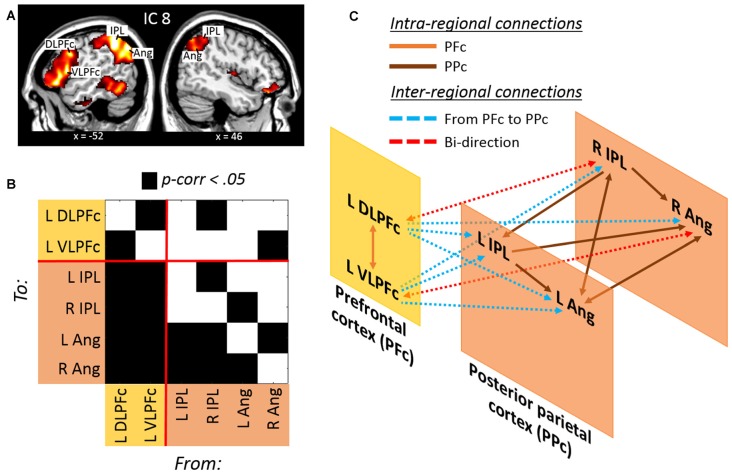
**(A)** Lateral prefrontal and posterior parietal regions derived from the IC no. 8 involved with divided attention across space. **(B)** Results of the Granger Causality Analysis (GCA) highlighting **(C)** a model of effective connectivity between prefrontal and posterior parietal regions. The model illustrates a prefrontal over posterior parietal control during divided attention across space (see the main text for further details).

Trials requiring dividing attention across the two hemifields involved the selective contribution of IC 8, as revealed by the analysis of beta weights that contrasted “att2mod > att2mod” (see Table 1). IC 8 included several regions of the posterior parietal cortex, extending from the inferior parietal lobule (IPL) to the angular gyrus (Ang), bilaterally. This component also included regions of the prefrontal cortex, but only on the left hemisphere, namely the left dorsolateral prefrontal cortex (DLPFc) and the left ventrolateral prefrontal cortex (VLPFc; see Figure 3A and Table 2).

The effective connectivity among these regions was investigated with GCA (Figure 3B and Table 3). Significant causal relationships were observed at both intra-regional (i.e., within the prefrontal and within the posterior parietal cortex) and inter-regional level (i.e., prefrontal/posterior parietal effective connectivity). This causal relationships are schematically illustrated in Figure 3C. Mutual causality was observed between the dorsolateral and ventrolateral regions of the prefrontal cortex. Similarly, an high level of mutual causality was observed within the posterior parietal cortex, although this was mainly driven by the left and right IPL, which were found to predict the activity of the left and right Ang. At the inter-regional level, the prefrontal regions were found to significantly predict the activity of the posteriors parietal nodes. In fact, both the DLPFc and the VLPFc showed causal relations with each single node of the posterior parietal cortex (see cyan lines in Figure 3C). By contrast, the posterior parietal cortex showed overall a reduced causal influence of the prefrontal nodes. This was evidenced by the fact that only the right IPL and the left Ang showed causal relations with the left DLPFc and the VLPFc, respectively, and that these two latter connections were bi-directional, i.e., not indicating any specific causal influence of the posterior parietal over the prefrontal nodes during trials requiring divided attention across different spatial locations.

**Table 3:** Holm-Bonferroni’s corrected *p*-values derived from the Granger causality analysis carried out among the nodes of the ICs no. 8 (see also Figure 3B).

To:	L DLPFc	—	0.000	0.090	0.000	0.350	0.195
	L VLPFc	0.000	—	0.456	0.209	0.456	0.008
	L IPL	0.000	0.000	—	0.000	0.063	0.448
	R IPL	0.000	0.000	0.116	—	0.000	0.090
	L Ang	0.001	0.000	0.000	0.021	—	0.000
	R Ang	0.004	0.000	0.000	0.000	0.000	—
		L DLPFc	L VLPFc	L IPL	R IPL	L Ang	R Ang
	From:						

#### Divided Attention across Sensory Modalities: ICA and GCA

**Figure 4:**
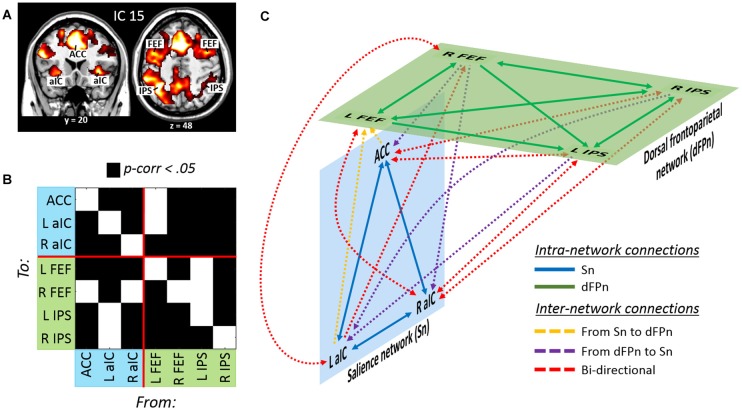
**(A)** Dorsal frontoparietal, anterior cingulate and insular regions derived from the IC no. 15 involved with divided attention across sensory modalities. **(B)** Results of the GCA highlighting **(C)** a model of effective connectivity between the nodes of the dorsal frontoparietal and the salience network. Overall, the model illustrates a tight interdependence between the two networks during divided attention across sensory modalities (see the main text for further details).

The analysis of beta weights revealed that IC 15 was selective involved with events requiring to divide attention across stimuli originated from different sensory modalities (“att2mod > att2loc”; see Table 1). IC 15 included regions belonging to the dorsal frontoparietal network (Corbetta and Shulman, 2002; Corbetta et al., 2008; Duncan, 2013), such as the FEF and the IPS, bilaterally. IC 15 also included regions belonging to the salience network (Menon and Uddin, 2010; Uddin, 2015), such as the anterior cingulate cortex (ACC) and the anterior insular cortex (aIC), bilaterally (see Figure 4A and Table 2).

As before, the effective connectivity among these regions was analyzed with GCA (Figure 4B and Table 4). Extensive intra- and inter-network causal relationships were observed, and these are schematically illustrated in Figure 4C. In the salience network, each of the three nodes showed mutual causal relationships with each of the other nodes. Similarly, mutual causality relations were observed among the nodes of the dorsal frontoparietal network, with the exception of the left IPS that did not predicted activity in the left and right FEF. At inter-network level, the GCA showed a number of mutual causal relationships between the dorsal frontoparietal and the salience network, highlighting a tight interdependence between these two networks. This was exemplified by the seven red lines in Figure 4C. By contrast, the GCA showed only a few uni-directional causal relationships from the dorsal frontoparietal to the salience network (two yellow lines) and from the salience to the dorsal frontoparietal network (three purple lines).

**Table 4:** Holm-Bonferroni’s corrected *p*-values derived from the Granger causality analysis carried out among the nodes of the ICs no. 15 (see also Figure 4B).

To:	ACC	—	0,000	0,000	0,454	0,000	0,000	0,013
	L aIC	0.000	—	0.000	0.454	0.000	0.000	0.000
	R aIC	0.000	0.000	—	0.006	0.000	0.000	0.000
	L FEF	0.000	0.000	0.000	—	0.000	0.369	0.000
	R FEF	0.052	0.000	0.086	0.000	—	0.158	0.000
	L IPS	0.021	0.136	0.000	0.001	0.000	—	0.000
	R IPS	0.000	0.136	0.000	0.014	0.000	0.000	—
		ACC	L aIC	R aIC	L FEF	R FEF	L IPS	R IPS
	From:							

## Discussion

The current study aimed at characterizing large-scale brain networks supporting divided attention by re-analyzing previously reported fMRI data (Santangelo et al., 2010). Consistent with standard fMRI analyses reported by the previous literature (Fagioli and Macaluso, 2009, 2016; Santangelo et al., 2010; Santangelo and Macaluso, 2013a), the current study found that divided attention was sustained by regions belonging to the frontal and parietal cortices. However, the current data-driven approach based on ICA and CGA also revealed novel findings compared to the previous literature. Specifically, important differences were observed depending on whether attentional resources were divided across different spatial locations or different sensory modalities, which is in agreement with the current behavioral data showing decreased performance when participants had to monitor both visual and auditory streams compared to when they had two monitor both the left and right hemifield.

As revealed by the ICA, dividing attentional resources across two spatial locations necessitated the recruitment of a brain network involving the left ventro- and dorso-lateral prefrontal cortex, plus the posterior parietal cortex, including the IPS and the angular gyrus, bilaterally (IC 8, Figure 3A). There is by now a large consensus about the key role played by the posterior parietal cortex in spatial-related tasks. In fact, the posterior parietal cortex has been associated with spatial attention (Corbetta et al., 2000; Yantis et al., 2002; Vandenberghe et al., 2005; Molenberghs et al., 2007; Kelley et al., 2008; Nardo et al., 2011, 2014), as well as with the storage of spatial information in working memory (Munk et al., 2002; Leung et al., 2004; Xu and Chun, 2006; Santangelo and Macaluso, 2013b; Santangelo et al., 2015; see also, for reviews, Zimmer, 2008; Santangelo, 2015). The lateral prefrontal cortex is instead thought to be deeply implicated in executive control (see, for reviews, Yuan and Raz, 2014; Koechlin, 2016) and to play a central role in strategic monitoring during working memory processes (see, for a review, Sreenivasan et al., 2014). While the posterior parietal cortex has been shown to be centrally involved with the maintenance of spatial representation, the activation of the lateral prefrontal cortex has been more related to the control of the information that is actually maintained in the posterior parietal cortex (Champod and Petrides, 2007). Consistently, several recent studies demonstrated that neural populations in the lateral prefrontal cortex can encode multiple task variables (Barak et al., 2010; Stokes et al., 2013), which allows high-dimensional representation of behavioral priorities. For instance, Machens et al. (2010) showed that single lateral prefrontal neurons contributed to the maintenance of multiple information, such as stimulus identity and elapsed time, but that each type of information can be independently extracted from the population code.

All this literature is consistent with the current findings showing the involvement of lateral prefrontal and posterior parietal regions during divided attention across spatial locations. The current data demonstrates for the first time in the context of divided attention that the posterior parietal regions are under controls of the ventro- and dorso-lateral prefrontal cortex. As revealed by the GCA, the prefrontal regions (i.e., the left DLPFc and the VLPFc) of IC 8 were found to predict the activity of all of the posteriors parietal nodes (see cyan lines in Figure 3C). Conversely, the causal influences of the posterior parietal nodes towards the prefrontal regions is rather limited, showing no uni-directional causal relationships with the prefrontal cortex. In agreement with the existent literature, the involvement of the lateral prefrontal cortex might be related to the effort of maintaining the current target representation, implemented by the posterior parietal cortex, on multiple spatial locations. The lateral prefrontal/posterior parietal mechanism might then pre-activate sensory cortices (McMains and Somers, 2004, 2005; Sreenivasan et al., 2014), thus enabling correct target detection among the continuous flow of audiovisual information across both hemifields.

The current findings demonstrated instead quite different neural resources implicated for monitoring multiple stimulus types/sensory modalities. As revealed by the ICA, dividing attention across different stimuli/sensory modalities necessitated the recruitment of IC 15. This brain network involved nodes belonging to the dorsal frontoparietal cortex (Corbetta and Shulman, 2002; Corbetta et al., 2008; Duncan, 2013), such as the FEF and the IPS, bilaterally, and the salience network (Menon and Uddin, 2010; Uddin, 2015), including the anterior cingulated cortex and the left and right aIC. The analysis of Granger causality highlights a tight interdependence between the dorsal frontoparietal and the salience network in trials requiring divided attention between different sensory modalities. In fact, most of the link between the two networks were bi-directional, indicating mutual causal relationships, which is in agreement with recent literature showing positive correlation between the frontoparietal and the salience network in a variety of tasks (see, for a review, Uddin, 2015). The current findings further extends the notion of frontoparietal/salience network co-variation in the domain of divided attention, showing that mutual interrelations between the dorsal frontoparietal and salience network might be fundamental to monitor concurrent stimuli coming from different sensory modalities. The salience network has key nodes in the aIC and ACC and is thought to be critical for detecting stimuli that are potentially relevant from a behavioral point of view (Menon and Uddin, 2010). Recently, the aIC has been shown to play an important role during multisensory attention. Chen et al. (2015) reported an fMRI study in which participants were asked to perform three different “oddball” tasks based on visual, auditory and auditory-visual stimuli. Chen et al. (2015) observed that the activity of the right anterior insula influenced the activity of all of the other emerging multisensory-related areas (i.e., frontal, cingulate and parietal cortex). The authors found that the role of the right anterior insula was more compatible with an “integrated signaling model” based on the simultaneous deployment of attention to both auditory and visual stimuli in the oddball task, rather than a “segregated signaling model” based on uncorrelated signals coming from each single sensory modality. What is more, this integrated model was particularly effective in accounting for the signals originating from the anterior cingulate and posterior parietal cortices, two important nodes of the salience and the frontoparietal network, respectively. These results were interpreted as an evidence that the anterior insula might serve as a control hub for the deployment of attentional resources on multisensory stimuli.

Consistent with Chen et al. (2015), monitoring simultaneous multisensory streams entailed here the recruitment of the salience network. However, the current data showed an interdependency between the salience and the dorsal frontoparietal network, more than a “control” role played by the salience over the dorsal frontoparietal network. This might be related to a key difference in task demands: while Chen et al. (2015) employed a task based on audio-visual integration (i.e., with audiovisual targets), in the current task participants were asked to monitor concurrent but separated audiovisual streams for detecting visual and auditory targets. The current requirement to monitor independent auditory and visual streams might have necessitated an increased interplay between the salience and the dorsal frontoparietal network.

To conclude, this study highlighted the key role played by the lateral prefrontal cortex in splitting attentional resources over multiple spatial locations, and by the salience network to divide attention towards multiple (visual and auditory) stimuli originating from the same location. Both the lateral prefrontal cortex and the salience network were shown to necessitate the contribution of different regions of the frontoparietal network during divided attention: dorsal frontoparietal regions (FEF and IPS) were linked to the salience network during divided attention towards audiovisual stimuli, while ventral regions of the posterior parietal cortex (IPL and Ang) were linked to the lateral prefrontal cortex during divided attention towards the left and right hemifield. The current findings therefore brought to light a dissociation between the brain networks implicated during divided attention across spatial location and sensory modalities, overall highlighting the importance of instigating the effective connectivity among large-scale brain networks supporting complex behavior.

## Author Contributions

VS conceived the study, collected and analyzed the data and wrote the manuscript.

## Conflict of Interest Statement

The author declares that the research was conducted in the absence of any commercial or financial relationships that could be construed as a potential conflict of interest.

## Supplementary Material

FIGURE S1 Discarded independent components.
