# Supplementary figures and images for: Large-Scale Brain Networks Supporting Divided Attention across Spatial Locations and Sensory Modalities
Source: Front Integr Neurosci. 2018 Feb 27;12:8. doi: 10.3389/fnint.2018.00008 (PMC5835354; doi:10.3389/fnint.2018.00008)

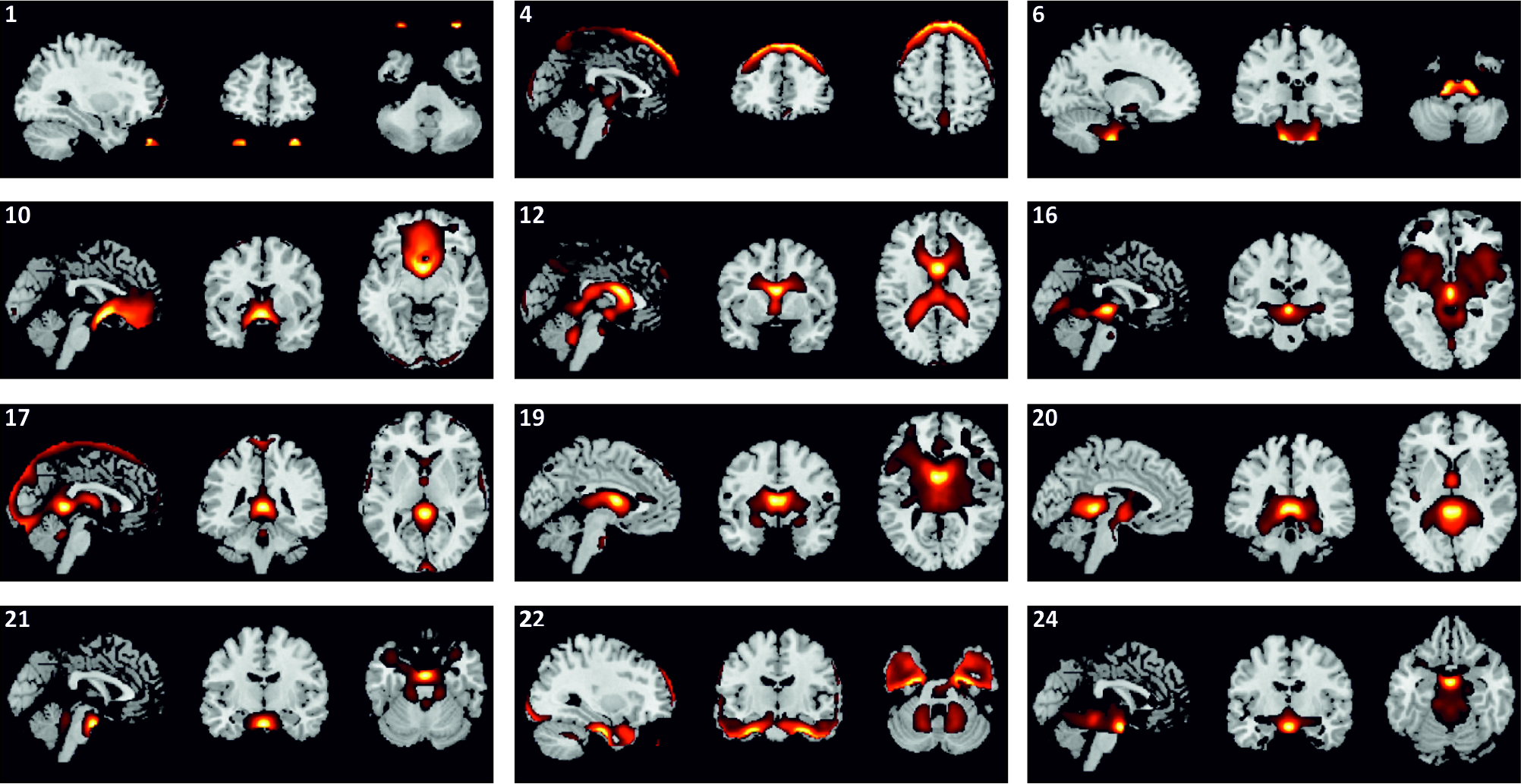

Supplement: FIGURE S1 — Discarded independent components. [file Image_1.tif]
